# Socioneuroscience and its contributions to conscious versus unconscious volition and control. The case of gender violence prevention

**DOI:** 10.3934/Neuroscience.2019.3.204

**Published:** 2019-09-10

**Authors:** Lídia Puigvert Mallart, Ramón Flecha García, Sandra Racionero-Plaza, Teresa Sordé-Martí

**Affiliations:** 1 Department of Sociology, University of Barcelona, Spain; 2 Centre for Community, Gender and Social Justice, Institute of Criminology, University of Cambridge, Cambridge, United Kingdom; 3 Department of Sociology, Autonomous University of Barcelona, Spain

**Keywords:** socioneuroscience, volition, control, brain plasticity, consciousness, unconsciousness, dominant coercive discourse, addicted consciousness and volition, social impact, gender violence prevention

## Abstract

Research in neuroscience is being very fruitful in providing evidence about the influence of social experience in the architecture and functioning of the brain. In so doing, neuroscience is posing new and fascinating research questions to examine in depth the social processes that produce those neural changes. To undertake the task of tackling such research questions, evidence from the social sciences are necessary to better understand how different types of social experiences produce different types of synaptic changes and even modify subcortical brain structures differently. It will be the dialogue between neuroscience, other natural sciences and the social sciences which will advance the scientific understanding of plastic changes in the brain which result from complex social experiences that have been traditionally studied by the social sciences. Socioneuroscience constitutes the arena for such interdisciplinary dialogue and research that can both advance the scientific understanding of the human brain and provide evidence-based solutions to most urgent social problems. Socioneuroscience studies the relations between the human brain and social interactions taking into account knowledge from all social sciences and the natural sciences. Processes of conscious versus unconscious social volition and control is one central area of inquiry in socioneuroscience. In this article, we discuss the *dominant coercive discourse* in society -which presents males with aggressive attitudes and behaviors as more attractive- as an example of social control of human volition which imprisons many individuals' sexual freedom. However, due to brain plasticity, certain experiences that question such dominant discourse and empty violence from attractiveness open up the possibility for the individual and the society to break free from the neural wiring imposed by the dominant coercive discourse and, in the words of Santiago Ramón y Cajal, be ourselves “the architects of our brain”, contributing to overcome violence against women.

## 1. Introduction: Social experience shapes brain architecture and gene expression

Research in neuroscience, both in animal models and in humans, has shed light on the influential role of social context and social experience in shaping brain architecture and function [1]–[3]. Eric Kandel, the Nobel Laurate in Physiology or Medicine in 2000, is clear in this regard. In his book “In search of memory” Kandel [4] states:

“Thus, even though I had long been taught that the genes of the brain are the governors of behavior, the absolute masters of our fate, our work showed that, in the brain as in bacteria, genes also are servants of the environment. They are guided by events in the outside world (p. 154).”

The hypothesis of brain plasticity, first time formulated by Santiago Ramón y Cajal [5], refers to the ability of synapses, neurons and entire brain regions to change their properties in response to use or to different profiles of stimuli [4]–[6]. The power of environmental stimuli to shape the brain in the case of humans has been well investigated in studies of twins, institutionalized children, and children victims of different kinds of abuse and neglect [5],[7],[8]. All such studies have provided strong evidence showing that because every human grows in a different environment and has different experiences, the architecture of each brain is different; in other words, each brain reflects the life story of an individual. Studies of twins with identical genes are among the most powerful in showing the influence of experience on the brain even beyond genetics; they demonstrate that those twin siblings show differences in brain architecture as a result of different social experiences and social interactions [4],[5],[9]. Scientific research in epigenetics has well advanced these findings, proving that the epigenome can be influenced by environmental factors, such as diet and toxic stress, and can end up producing phenotypes and be inherited [10],[11]. These modifications can occur at any time in development, yet the period of embryonic development is particularly sensitive.

This capacity of the human brain to construct and reconstruct itself via social experience can occur in positive and negative directions. While adverse social experiences have shown to damage brain architecture, positive social experiences can reverse such harm significantly and, in the absence of adversity, to support strong brain development throughout life. Studies in cognitive and affective neuroscience have shown that adverse social relationships produce toxic stress; such stress hinders brain development [1] and worsens health outcomes [2]. Exposure to stressful experiences has been shown to alter the size and neuronal architecture of the amygdala, the hippocampus, and the prefrontal cortex (PFC) [12], as well as toxic social experiences alter learning, memory, and executive functioning [1]. When such experiences are not exceptional but persist for a long period of time, the consequences are more negative. Animal and human studies have shown that persistently elevated levels of stress hormones (cortisol, adrenaline) can disrupt the developing architecture of the brain [1]. High levels of adversity can even produce loss of neurons and neural connections in the hippocampus and medial prefrontal cortex, which will affect the subject's memory and complex cognition. The biological manifestations of toxic stress can include alterations in immune function and appreciable increases in inflammatory markers, which are known to be associated with poor health outcomes as varied as cardiovascular disease, viral hepatitis, liver cancer, asthma, chronic obstructive pulmonary disease, autoimmune diseases, poor dental health, and depression [13],[14].

Despite brain plasticity is conserved throughout life, evidence also indicates that there are times in development that are particularly sensitive for the brain to be modified by experience. We now know that the earlier severe stress is experienced, the worse the impact in brain architecture. For instance, recent neuroscience research has indicated that when very adverse social relationships, in particular, severe maltreatment and neglect, are experienced the first five years of life, the greater is the likelihood for reduced hippocampal volume [12]. This subcortical modification has important implications for the survivors' later life and may include heightened vulnerability to brain and behavior disorders following trauma, poorer antidepressant response, and memory deficits [12]. Another recent study [15] has shown that early life adversity is associated with increased gastrointestinal symptoms in children that may have an impact on their brain and behavior as they mature. This evidence is transforming the clinical practice for gastroenterologists and other health-care providers, as for when and how to inquiry about a history of trauma and abuse, and what to do with that information for the implementation of most proper care of the patients [16].

Importantly, the same brain plasticity applies to understand that the possibility to experience such negative implications in youth and adulthood will be influenced by the type of social experiences that the person will have, whether they are again adverse or protective. This leads us to the positive side of plasticity. Research in neuroscience has shown that positive social experiences support healthy brain architecture [1],[3], and quality human relationships have been shown to be one of the most influential factors for such positive brain development and better health outcomes [17],[18]. The Harvard Longitudinal Study on Adult Development has shared the best available evidence in this regard [19],[20]. A longitudinal study of 80 years of duration conducted upon two male cohorts (N = 724), one of Harvard students and one of youth from poor neighborhoods in Boston, collected since 1938 various types of data in every subject, including blood tests, brain scans, follow up about their employment and professions, income, interviews and surveys about their social relations in the family and the community, diet and exercise habits, etc. The results were strong: among all the factors explored, it was close human relationships of different kinds, in the community, friendships, in the family, etc, the ones which predicted most longer, healthier and happier life above and beyond factors such as socioeconomic status, QI and even genes. It was quality relationships the ones that protected the individuals from crises of meaning in life and helped them delay the decay in mental and physical health [19].

In the last decade, many other research projects in affective and cognitive neuroscience have come to add important evidence in the same line, connecting better relationships with better brain functioning and better physical and mental health outcomes. For example, a study [21] has proven that viewing pictures of a romantic partner reduces experimental pain because the image induces recall of good memories with that person, and this, in turn, elicited positive emotions. The relief in the pain experimented while viewing images of the romantic partner is associated with the activation of neural reward systems. Such relief effects have been compared with the effects of anesthesia. Also, fMRI studies comparing superficial versus quality peer relationships in adolescence [3] have evidenced that one or the other type of friendships shape neural reward systems differently. Adolescents in high quality relationships show less sensitivity in neural reward systems (ventral stratum and insula), so risk taking behavior becomes less rewarding and less meaningful. Differently, adolescents in superficial peer relationships show greater sensitivity in their neural reward systems so that risk behaviors become to them more attractive and meaningful. This implies that the quality of adolescents' peer relationships modulates neural sensitivity to risk taking [3].

## 2. Socioneuroscience for the scientific understanding of the bidirectional relations between the human brain and social interactions. Differences with social neuroscience

The strong evidence on the important role of social experience, human relationships, and social interactions in influencing brain architecture sheds light on the relevant role for social sciences in neuroscientific studies. The body of evidence accumulated in the social sciences is of major relevance to be able to better understand the very complex relations between social experiences and neural development. *Socioneuroscience* [22] takes into account scientific knowledge from all social sciences and all the natural sciences to study the relationships and interlinks between the human brain and human interactions. The field is clearly separated from the remit of social neuroscience.

Whereas social neuroscience [23] examines how the brain mediates social processes and behavior, socioneuroscience studies the bidirectional relations between the brain and human interactions. The analyses from socioneuroscience involve taking into account how the brain mediates social interaction and social behavior, but not only, as it is mainly crucially concerned with how different types of social interactions and social experiences can shape the brain in different directions with positive or negative outcomes for the individual and the society.

While social neuroscience explores how the biology of the brain influences social processes, including social interactions, prejudice, agency, empathy, affiliations, and morality among others, socioneuroscience explores a wide range of human interactions and pressing social problems involving social relationships, but from the perspective of the bidirectional relationship between the brain and human interactions. In November 2009, at the Society for Neuroscience meeting, “Social neuroscience was defined broadly as the interdisciplinary study of the neural, hormonal, cellular, and genetic mechanisms underlying the emergent structures that define social species” [24]. Socioneuroscience is not a biological account of how the brain mediates particular social interactions but the analysis of human relationships using knowledge from all sciences, natural and social, starting from the existing scientific evidence, also in neuroscience, that shows that social experience mediates brain development, modifies synaptic activity and even shapes brain architecture.

The difference between social neuroscience and socioneuroscience also relies in the application of concepts and methods. Social neuroscience mostly applies concepts and methods of biology to develop theories of social processes and behavior in the social and behavioral sciences, and sometimes uses social and behavioral constructs and data to advance theorical explanations of neural organization and function. Socioneuroscience applies theories and concepts from all the natural and all the social sciences to develop multidisciplinary theories about how the brain and human interactions relate. This difference leads to a distinction in method. While social neuroscience mostly applies neurobiological techniques including functional magnetic resonance imaging (fMRI), electroencephalography (EEG), event-related potentials (ERPs), magnetoencephalography (MEG), positron emission tomography (PET), facial electromyography (EMG), transcranial magnetic stimulation (TMS), electrocardiograms, galvanic skin response (GSR), electromyograms, single-cell recording, etc, socioneuroscience uses such techniques as well as methods in the social sciences, including qualitative research (interviews, focus groups, life stories, etc) and mixed methods research, also combining all qualitative methods and mixed methods with the use of neurobiological techniques.

Socioneursocience is in line with what Eric Kandel [25] announces as a new scientific humanism, “a new humanism, one that merges the sciences, which are concerned with the natural world, and the humanities, which are concerned with the meaning of human experience” (p. 13). Socioneuroscience brings the natural sciences and the social sciences into egalitarian dialogue in their joint endeavor of providing better scientific accounts to all citizens about their human nature and society as a tool for them to better understand themselves and guide their behavior and development not only as individuals but as civilization in history. Socioneuroscience can ultimately contribute to informing actions and strategies that support a healthier and more meaningful life for all citizens as well as more democratic societies.

Among the social sciences, sociology has examined for more than two centuries now how social structure and, more particularly, social, cultural and economic inequalities influence and shape society and the social behavior of individuals and groups; even classical sociologists deepened into the crisis of meaning [26] which emerges as a rupture of the unity between beauty, goodness, and truth. Interactionist [27], communicative [28], and dialogic [29] sociology has provided in-depth analyses about how communicative interaction relates to social structure and can also challenge it, and how different types of communicative acts can enhance democracy or increase violence in social relations and in society [30]. Mead's [27] described the dialogic self, where society is within the individual through the “Me”, Habermas [28] pointed to communicative action and communicative rationality as the ones which can produce mutual understanding among subjects, and the theory of communicative acts [29] has shed light on the importance of attending to inequalities and power relationships in social structure as well as to non-verbal behavior to differentiate between *dialogic communicative acts* and *power communicative acts* in communicative interaction.

The consideration in neuroscience of all these social theories and research studies inform a new scientific sight into the human brain, a sight informed with the nuances, complexities and aspirations of social life in everyday settings. The utility of scientific research which undertakes this socioneuroscientific sight can be very high to provide profound evidences that can aid solving most pressing social problems. For example, the differentiation between dialogic and power communicative acts in the theory of communicative acts [30] is essential to understand how different types of communication in social interaction can produce very different results, not only in terms of mutual understanding or not, but also dialogic and power communicative acts can trigger very different thoughts, feelings and emotional reactions that either support or weaken brain functioning and architecture. The theory of communicative acts proofs that coercion (which implies adversity) might result from communication between two subjects not because of the speech (what is said) but because of the social structure which frames the interaction and other non-verbal behavior, including gestures and gazes. As Soler [30] explains, inviting a woman for a coffee after work has no negative note in itself. However, if such invite is from a boss and the woman is her employer, then the characteristics of the interaction might produce coercion because such interaction is framed by an unequal social structure which implies unequal distribution of power where the boss is in a position of power over the female employee. In the presence of coercion, such social interaction is adverse, in the sense also taken by neuroscience. On the contrary, if the interactants, particularly those holding more power, are aware of the unequal social structure and its influence on the interaction, and are responsible for its potential consequences, then they can act in ways that control such structural factors and ensure greater freedom in the relationship. If so, such social interaction is not only non-adverse but it can be enriching and meaningful for both subjects.

Likewise, the social sciences have well investigated poverty, terrorism, crime, war, racism, sexism, and many forms of inequality, exclusion and violence, paying particular attention to vulnerable groups, such as cultural, ethnic and religious minorities, women, youth, people with disabilities, the poor, immigrants and refugees, etc. Those environments of inequality, and relationships and contexts that involve violence and hate [31],[32] are the ones that neuroscience has indicated to contain toxic stressors that weaken brain development and architecture [1],[2],[7],[8]. Research in psychology has studied such environments in depth, accumulating a large body of evidence on the mechanisms by which toxic and violent relationships, such as gender violence and bullying, deteriorate the mental health of the victims [33],[34]. Preliminary findings from psychoneuroendocrinology research suggest that victims of Intimate Partner Violence and women traumatized by childhood abuse may suffer from alterations in hypothalamic-pituitary-adrenal axis functioning [35]. Studies in the areas of gender studies and criminology have also provided scientific accounts on factors favoring gender violence victimization and sexual harassment [36], as well as have identified effective preventive and response strategies [37].

In taking this accumulated knowledge on exclusion and violence from the social sciences into account, together with knowledge from the health sciences and other natural sciences, socioneuroscience research can provide more nuanced scientific explanations on the impact of violence and discrimination on brain architecture, and doing so for the most pressing social problems that societies worldwide need to address and solve, ranging from gender violence and sexual harassment to violent radicalization.

At the same time, research in social sciences increasingly addresses the identification of what has been termed as *successful actions*, those evidence-based actions that have common universal characteristics and are transferable to diverse settings producing transformation in contexts of adversity [38]. The existing evidence on positive outcomes produced by successful actions in health [39], education [40],[41], gender [42], employment and economy [43], and political participation [44] among other fields, implies new venues for socioneuroscience research to shed light on whether and how successful actions in those social areas constitute experiences that can have a positive impact also in brain architecture, buffering the effects of socio-economic and health adversity in the brain.

In all, the combination of the large body of evidence in social sciences and in the natural sciences can raise cutting-edge interdisciplinary research questions that provide more nuanced scientific explanations on brain plasticity and ultimately advance the comprehension on how the human brain and social interactions relate. From the point of view of social impact of science [45], such integration of interdisciplinary knowledge can provide a stronger evidence-base for transformative interventions.

## 3. Contributions from socioneuroscience to conscious versus unconscious volition and control: The case of the dominant coercive discourse

The overcoming of violence against women is one pressing issue worldwide. Epidemiological data informs that 35% of women worldwide have experienced either physical and/or sexual intimate partner violence or non-partner sexual violence [46]; 13 million women in the EU had experienced physical violence in the course of 12 months before the survey interviews conducted for the EU Agency for Fundamental Rights [47]. The same European survey showed that one in three women had experienced physical and or sexual violence since they were 15 years old, and out of all women with a (current or previous) partner, 22% had experienced physical and/or sexual violence by a partner since the age of 15. Regarding non-partner violence, 22% has experienced physical violence by someone other than their partner since the age of 15. Gender violence is a problem affecting women in all cultures and countries, and a trend of increasing victimization among female adolescents and preadolescents is of major concern [48]. The social sciences and the health sciences have studied violence against women for decades, providing explanations for its causes and consequences, as well as pointing to measures of prevention and response [49].

Research in psychology, gender studies, sociology, and criminology about preventive socialization of gender violence [50] has shed light on one risk factor for gender violence victimization. These studies have pointed to the existence of a *dominant coercive discourse* in society [51] which, shaped by an imbalance in power within relationships, influences socialization into linking attractiveness to males with violent attitudes and behaviors, while non-violent men and relationships are -because of this coercive dominant discourse- mostly perceived as convenient but not exciting [52]. This discourse is learned though socialization, in which the media and social interactions play a crucial role [50]. After encountering this discourse in numerous repeated occasions in socialization agents and in conversations with peers, friends, and others, the association between attractiveness and violence gets internalized by the individual, shaping own's cognitive and affective schemata, up to a point in which such learning (the discourse) can drive thoughts, feelings and behaviors in almost unconscious ways. Therefore, a woman can see a man who illustrates such dominant traditional masculinity with aggressive attitudes and behaviors and feel attraction toward him, with subsequent physiological responses such as accelerated beat, greater arousal, major attention, and other reactions in various systems of her organism. Those are emotional reactions triggered by certain environmental stimuli [5], stimuli which have the meaning imposed by the coercive dominant discourse.

This learning of the dominant coercive discourse follows the logic which has been explained in memory studies in cognitive psychology [53]–[55] and neuroscience [4],[5]. Such research has evidenced that the transfer of information from short term memory to long term memory depends mainly on rehearsal, repetition and practice. Repeated social experiences lead to stronger neural connections (long term memory), and the stronger the synaptic connections, the more likely less conscious responses [4],[5]. It is expected then, that the more a person socializes into the coercive dominant discourse -through indirect and direct exposition, and via experiences of sporadic or stable relationships with such aggressive men- the more likely the development and tenacity of the neural circuity which is the home of the dominant coercive discourse. Therefore, when a woman socialized into the dominant coercive discourse has emotional reactions of attraction that she can feel in her body and mind when seeing, interacting or remembering a relationship with a man with aggressive or violent behavior, those physiological reactions are the result of the social control played by the dominant coercive discourse. Far from such emotions and feelings being their “own”, those are controlled and imposed by an external discourse that is shaped by the patriarchal system.

Importantly, the dominant coercive discourse learned affects not only some women's current sexual-affective thought and behavior, but it might also influence how past sexual-affective experiences are remembered and interpreted [56]. That is, the dominant coercive discourse can also control the cognitive and emotional interpretation of autobiographical memories of violent sexual-affective relationships, this implying the possibility of feeling some degree of attraction when recalling the violent relationship and the aggressive man in it. This is of fundamental relevance from the point of view of human development and health. Psychological studies on autobiographical memory [57] have well demonstrated that one of the most important functions of autobiographical memory is prospective, that is, it aids planning future acts: we storage information about our most meaningful personal experiences in the service of planning for the personal future [58]. Depending on the meanings and feelings attached to those memories, then our future plans will take different directions, more or less healthy and meaningful. In the case of memories of violent sexual-affective relationships, because of the control of the coercive dominant discourse over them, they can be imbued with feelings of attraction. Research in social sciences has already demonstrated this [56],[59].

Flecha, Puigvert and Ríos [59] report the case of a 15-year-old female adolescent who shares her memories of her first sexual experience, which was in the context of a sporadic sexual relationship. In her oral reporting, she acknowledged that the man forced her to have sex, he imposed the how and where, as well as she shared it was very painful. There was no sexual pleasure. Yet the young woman remembers that after that violent experience, she was in love with that man who did not want to see her again:

“When I was 15 years old, the first relationship I had he forced me. (…), I wanted to be liked by him. (…) I made it very easy for him. So he decided that he would do me on a beach, I remember that I had never done it and I was very nervous, it was really painful. After that I cried a lot because it was really painful… And I remember he never talked to me again (…) So yes, there was no sexual pleasure, it was a lot of pain... And after that I was so in love with this guy, but he didn't want to be with me never again (p. 96).”

This is a violent social relationship which involves toxic stressors, among others, sexual abuse and psychological violence. This adverse experience is stored in long term memory and, as shown in the example, the memory involves at the same time consciousness of maltreatment and feeling of attraction; that is the association established by the dominant coercive discourse.

However, as neuroscience has stated, what is uniquely human are feelings, not emotions [5]. Whereas emotions are the physiological reactions which happen more or less unconsciously when the brain detects defiant situations, feelings are the conscious experience of those somatic and cognitive changes, i.e., the conscious perceptions of emotional experiences [5]. While emotional responses are found in very simple organisms, such as bacterial cells, feelings are a very human characteristic because consciousness is what mostly defines humans.

The ability of humans to reflect upon and about themselves -who I/we am/are and who I/we want to be-, self-consciousness, opens a window for transformation of cognitive and affective schemata and memories to ultimately break free from the control of the dominant coercive discourse on individual sexual-affective desire. It is through critical awareness about the control of such social discourse in ourselves, our emotions, feelings and behavior, how individuals can, if they decide to do so (volition), empty memories of violent sexual-affective relationships of any attraction and, more generally, disassociate attractiveness from aggressive attitudes and behaviors. Socioneuroscience explores the social experiences and social interactions that are more effective in favoring this freeing process.

Research conducted with women between 17 and 30-year-old who had experienced violent sexual-affective relationships, mostly sporadic ones, has shown the ability of a very single intervention to favor raising critical consciousness in the participant subjects about the influence of the coercive dominant discourse in those intimate experiences [56]. The intervention design reflected the idea of brain plasticity as expressed by Kandel [4]:

“If you remember anything of this book, it will be because your brain is slightly different after you have finished reading it. This ability to grow new synaptic connections as a result of experience appears to have been conserved throughout evolution (p. 162).”

Women in the experimental condition of that study read three chapters of the book “Radical Love: A Revolution for the 21^st^ Century” [50], which discusses the dominant coercive discourse in society and its impact in individuals' sexual-affective cognitive and affective schemata. Before and after the reading, the participants wrote their memories of the violent sexual-affective relationship they had chosen to share. They also completed a memory quality questionnaire [60], mainly focused on emotions triggered by the recall. The reading favored participants to revise their memories with new lenses. Via critical consciousness, the participants reconstructed their memories of the violent relationships reported, both at semantic and emotional levels. At the semantic level, they increased the critical episodic details recalled in those memories -that is, violent details of the relationship-. At the emotional level, and crucially, they changed the emotions induced by the recall in the direction of fostering major rejection of the violent relationship and of the man in it [56]. As explained by some of the participants in the communicative discussion groups organized after the post-test, the revision of their memories did not take place in isolation, but some of the young women engaged their friends in the process, sharing paragraphs of the reading with them via face to face and mobile apps, thus giving a collective dimension to memory reconstruction and opening up possibilities for their friends to also reconstruct similar memories.

In those discussion groups after the intervention, some participants shared with the researchers evidence of the social impact of the research. Some of the young females informed that they had changed their minds about future sexual-affective relationships, which they now imagine to be very different from the ones shared in the study; they meant relationships completely free from any type of violence. Also, one participant left the violent relationship in which she was at the moment of the intervention; she shared that her personal experience with the intervention was a crucial drive to make that decision [56].

The results of this intervention research show that certain social experiences, such as reading a particular book, can make subjects aware of their *addicted consciousness*, a consciousness addicted to the dominant coercive discourse. Through critical awareness and quality relationships, subjects can question and modify the cognitive and affective schemata imposed by the dominant coercive discourse, and thus think, feel, and choose in their intimate life by their own volition, and no by a *volition* also *addicted* to the dominant coercive discourse. In the words of Santiago Ramón y Cajal, when subjects do this, they become architects of their own brain, a possibility that we, all humans, have. For the case discussed here, and because of the prospective function of autobiographical memories [61], the reconstruction that the participant subjects made of their memories of violent sexual-affective relationships increased their refusal to engage in stable or sporadic violent sexual-affective relationships and thus the likelihood to enjoy quality intimate relationships. If this actually occurs, new windows get open for a more meaningful and healthier future in young women victims of gender violence. The meaning of this potential impact is extremely profound.

Research [62] in neuroscience has shown that being in a romantic relationship - which is by definition opposed to any kind of violence - is associated with structural alterations in the human brain and that that individuals in a romantic relationship have enhanced perceived subjective happiness, all resulting from positive experiences. Research in psychology and health sciences has shown that people in secure, committed romantic relationships have a lower risk of heart disease, while those who have relationship discord have a higher risk [63]. Also, individuals in romantic relationships show lower ambulatory pressure, better immune functioning, and higher plasma oxytocin levels, the hormone related to feelings of happiness and wellbeing [64]. Additionally, psychological studies have proven that in romantic pairs in which a member suffers from chronic pain, emotional disclosure and validation by the other member can attenuate pain responses [64]. Research in criminology and gender studies has indicated the high presence of violence victimization in sporadic sexual relationships [35]. All these data has led to consider romantic relationships as one type of quality human relationships that can be life-saving [17].

All research in socioneuroscience that advances this knowledge on the power of romantic or ideal love to support healthy brain architecture and better health outcomes dismantles false ideas in society such as that romantic love kills [65],[66], a fabricated idea in line with the coercive dominant discourse which is pushing very young women to violent sexual-affective relationships, with all the very negative consequences that toxic relationships have for their brain and health as already reviewed in this article.

## 4. Conclusion: A new scientific and human horizon ahead

Neuroscientific research has provided strong evidence on the influence of social experiences in brain architecture. Most research in this regard has focused on the negative effects of adverse contexts and toxic stressors in the brain which deteriorate cognitive functioning and can even reduce the volume of some subcortical structures. More recent neuroscientific studies have also proven the benefits of positive contexts and human relationships for neural development. All this evidence in neuroscience dismantles the idea that biology is the basis of the human being, but indicates that the ability of social experience and social interaction to shape the human brain and biology in either healthy or unhealthy directions is remarkable. This very finding makes clear the centrality of studying the complex relations between the human brain and social interactions and the important role that social sciences have in such scientific endeavor. In this context emerges socioneuroscience, that undertakes the study of the relations between the human brain and social interactions taking into account knowledge from all social sciences and all the natural sciences.

One key central question in neuroscience has been conscious versus unconscious volition and control. Socioneuroscience research can provide a new sight on this topic. In this article, we have illustrated this with the case of the impact of the dominant coercive discourse [51] in society -a cause for gender violence victimization-, on individual volition and control. Through socialization, that is, social experiences with the media and via social interaction, many young women learn the coercive discourse, which shapes their cognitive and affective schemata. Through repeated experience, the coercive discourse, together with its corresponding neural connections, gets reinforced. This reinforcement can produce more automatic or unconscious emotional responses, so that in the face of the stimuli, i.e., aggressive male behavior and attitudes, the feeling of attraction can be triggered. In such situation, both the individual's consciousness and her or his volition are controlled by the coercive social discourse, and both consciousness and volition are therefore addicted to it. However, as illustrated in studies of the line of research on preventive socialization of gender violence [56],[67], all humans have agency, critical awareness that can be used to question such control of the dominant coercive discourse. Certain social experiences and social interactions can favor a context for such questioning to occur. We have shared here the instance of reading the book *Radical Love* [50] as an experience that aid young women's reconstruction of memories of violent sexual-affective relationships in a direction that supported their rejection of the aggressive behavior of the man in the relationship and of toxic intimate relationships more generally [56]. The social impact of the intervention study involved some participant women to transform their prospective thinking regarding future sexual-affective relationships, and one subject abandoned a violent sexual relationship. Scientifically, the results achieved show that certain social experiences can debilitate at the cognitive level the dominant coercive discourse learned, in ways that consciousness and volition end being addicted to that discourse and the individual can freely choose upon her or his sexual-affective desires, thoughts, and behavior.

In terms of future research in socioneuroscience, new studies could advance results of this and related research by including, for example, the brain scanning of the participants before and after the evidence-based intervention. In so doing, not only we can reach deeper understanding of the neural networks involved in sexual-affective attraction and desire addicted to the coercive discourse, but how that particular intervention modifies, if any and if the subject applies conscious volition to do so, such networks for the good. This analysis focused on brain plasticity could also be undertaken in a similar intervention research but where the reading of *Radical Love* is not individual but collective, discussed in group following egalitarian dialogue among participant subjects. Given the accumulated knowledge on the role of social interaction and dialogue in memory reconstruction [57], and the transformative power of dialogic feminist gatherings [67], it is expected this new interactive design can have different and maybe greater impact in the prevention of gender violence victimization and also in related brain architecture. This study is now in its initial phase.

Rita Levi-Montalcini, the Nobel Laurate in Physiology or Medicine in 1986, stated that “progress depends on our brains. And the most important part of our brains, the one which is neocortical, must be used to help others and not only to make discoveries”. Socioneuroscience opens up the possibility for scientists in all disciplines to advance scientific knowledge on the connections between the social realm and the human brain to support better life and a better world for us and for the generations to come.
